# The Effect of the Dose of Isotonic Saline on the Correction of Serum Sodium in the Treatment of Hypovolemic Hyponatremia

**DOI:** 10.3390/jcm9113567

**Published:** 2020-11-05

**Authors:** Jorge Gabriel Ruiz-Sánchez, Diego Meneses, Cristina Álvarez-Escolá, Martin Cuesta, Alfonso Luis Calle-Pascual, Isabelle Runkle

**Affiliations:** 1 Servicio de Endocrinología y Nutrición, Hospital Clínico San Carlos, Instituto de Investigación Sanitaria del Hospital Clínico San Carlos (IdISSC), 28040 Madrid, Spain; cuestamartintutor@gmail.com (M.C.); acallepascual@hotmail.com (A.L.C.-P.); irunkledelavega@gmail.com (I.R.); 2 Departamento de Endocrinología, Hospital Universitario Fundación Jiménez Díaz, 28040 Madrid, Spain; samoth67@gmail.com; 3 Servicio de Endocrinología y Nutrición, Hospital Universitario La Paz, 28046 Madrid, Spain; escola.cristina@gmail.com; 4 Centro de Investigación Biomédica en Red de Diabetes y Enfermedades Metabólicas Asociadas (CIBERDEM), 28029 Madrid, Spain

**Keywords:** hyponatremia, hypovolemic hyponatremia, hyponatremia treatment, overcorrection, isotonic saline, physiological saline

## Abstract

Background: Overcorrection of serum sodium (SNa) during therapy of hyponatremia can result in osmotic demyelination syndrome. Our aim was to determine the relationship between the isotonic saline solution dose (ISSD) administered and the 24-h SNa increase (24SNa) in patients with hypovolemic hyponatremia (HH). Methods: Retrospective study of HH patients treated with ISS in a tertiary hospital of Madrid, Spain, between 1 January–30 May 2019. The 24-h ISSD received and corresponding 24SNa were calculated. The latter was classified as 3 groups: ≥8 mmol/L, ≥6 mmol/L, or <4 mmol/L. Multivariate regression analyses were performed and ROC curves calculated to study the relationship between ISSD and 24SNa. Results: Thirty patients were included, age 72 years (60–80), 50% were women. 24SNa was ≥8 mmol/L/24 h in 33%, ≥6 mmol/L/24 h in 50%, and <4 mmol/L/24 h in 30%. Median ISSD in each group was: 32 mL/kg/24 h (29–37), 31 mL/kg/24 h (25–33), and 20 mL/kg/24 h (14–22), respectively. An ISSD ≥ 30 mL/kg/24 h had an odds ratio (OR) of 16 (95% CI: 2.5–95.1; *p* = 0.004) for a 24SNa ≥8 mmol/L, with a sensitivity and specificity of 80%. Conclusions: The 24SNa depends on ISSD. An ISSD between 23–30 mL/kg/24 h seems to be safe and effective.

## 1. Introduction

Hyponatremia is the most frequently encountered electrolyte disorder in clinical practice [1,2,3]. Patients with hypovolemic hyponatremia (HH) are characterized by a reduction in total body water. Effective circulating volume (ECV) is reduced, inducing non-osmotic stimulation of arginine vasopressin (AVP) secretion via baroreceptors [1]. In fact, reductions of ECV as low as 5% stimulate AVP secretion [4], inducing antidiuresis. The stimulus is acute, and hypotension in experimental animals can result in the liberation of AVP from the neurohypophysis within 1 min [5,6]. Conversely, the correction of hypovolemia will rapidly inhibit AVP secretion [4], reestablishing diuresis and increasing serum sodium (SNa).

Mild/moderate HH is usually treated with IV infusion of an isotonic saline solution (ISS) [1,2]. ISSs are crystalloid solutions containing from 131 to 154 mmol/L of sodium [7,8]. This therapy has often been associated with marked increases in SNa during the first 24 and/or 48 h of infusion [1,2,9,10]. High SNa increments have been found to facilitate the development of osmotic demyelination syndrome (ODS), a condition with an elevated morbidity and mortality. Patients with chronic hyponatremia, lasting a minimum of 48 h, are at particularly high risk for the development of ODS when presenting risk factors such as alcoholism, liver disease, hypokalemia, and/or malnutrition [11,12,13,14]. Although the goal in the treatment of patients with chronic hyponatremia is a 24-h SNa rise of 4–6 mmol/L [1,2], guidelines have defined overcorrection of hyponatremia as a 24-h elevation of SNa (24SNa) >10 mmol/L [1] or > 10–12 mmol/L [2] in patients with chronic hyponatremia, or over 8 mmol/L in patients at a high risk for ODS. SNa increments higher than 18 mmol/L in 48 h after initiation of therapy are also defined as overcorrection. In patients with risk factors for ODS, SNa should not increase by more than 8 mmol/L in each 24-h period during the first 48 h [2].

The relationship between the dose of ISS (ISSD) used to treat HH and the risk for overcorrection of SNa has yet to be elucidated. For this reason, our goal was to evaluate the relationship between the ISSD administered and the 24-h SNa response in a group of patients with HH, in an attempt to ascertain what would constitute a “safe” and effective dose.

## 2. Experimental Section

The current study is a retrospective analysis of a consecutive series of patients over 18 years of age, with HH, treated by clinicians of the endocrinology department of La Paz, a tertiary hospital in Madrid, Spain. Therapy consisted of IV infusion of ISS (NaCl 0.9%, or a balanced isotonic crystalloid solution of NaCl 0.81%) for the correction of HH. Treatment was initiated either in the emergency room, or on the wards. Patients were followed up daily for a minimum of two consecutive days. The patients were treated between January 1st and May 30th of 2019.

Patients were diagnosed with HH on the basis of (1) SNa <135 mmol/L (after correction for glycemia) [15], (2) maximum height of the internal jugular pulse (HIJP) less than 1 cm over the sternal angle with the patient reclined at 0–30° [16], and (3) at least two of the following: thirst, orthostatic symptoms, hypotension (blood pressure ≤90/60 mmHg), tachycardia (heart rate ≥90 bpm), urinary sodium (US) ≤ 30 mmol/L [17,18], or a rise in serum creatinine/urea accompanying the descent in SNa [19,20]. When the HIJP was not measured, HH was diagnosed with the presence of at least three of the other clinical features previously described above. All HH patients with serum biochemical parameters measured exactly 24 h after ISS infusion were included. No patient with a prior history of heart failure or a clinical history or physical examination compatible with heart failure was included.

Serum electrolytes were measured by indirect electrode methodology. Blood and urine samples were collected for biochemical analysis at baseline, as well as 24 h after the initiation of ISS therapy. Baseline variables evaluated were: age, sex, recent weight, SNa (with a SNa ≤120 mmol/L considered severe hyponatremia), serum potassium (SK), serum creatinine, serum urea, serum glycemia, the duration of hyponatremia prior to initiation of therapy (classified as acute hyponatremia if <48 h, and chronic if ≥48 h), risk factors for the development of ODS (SNa <105 mmol/L, malnutrition, hypokalemia, alcoholism, liver disease), and the setting in which therapy was initiated (emergency room, medical or surgical ward).

Data collected 24 h following initiation of ISS therapy were: ISSD administered IV in mL/kg body weight/24 h, SNa, SK, serum creatinine, serum urea, glycemia, etiology of hypovolemia (gastrointestinal losses—vomiting and/or diarrhea, urinary losses—diuretic therapy or US >30 mmol/L-, hemorrhage, or indeterminate if the prior causes were not present), type of ISS infused (NaCl 0.9% (containing 154 mmol/L of Na^+^) or balanced crystalloid NaCl 0.81% (containing 140 mmol/L of Na^+^), both considered isotonic solutions [7,8]), oral or IV therapy with potassium chloride (KCl).

The main variable studied was the 24SNa increment following initiation of ISS. Patients were classified as a function of the 24SNa rise as follows: ≥8 mmol/L (∆ ≥ 8), ≥6 mmol/L (∆ ≥ 6), or <4 mmol/L (∆ < 4). The limits used were chosen based on guideline goals for SNa correction in chronic hyponatremia [2,21,22], as well as the upper limit in patients with risk factors for ODS. Insufficient therapy or undertreatment was defined as a 24SNa increment below 4 mmol/L (∆ < 4).

The descriptive analysis of the categorical variables was carried out with frequencies and percentages, and that of the quantitative variables with measures of central tendency. Non-parametric variables expressed as the median (interquartile range: IQR) were used. Comparative analysis of the quantitative variables was performed using Mann-Whitney U or Kruskal-Wallis tests. The chi-squared test and the Fisher test were used for qualitative variables. Boxplot graphics were used to show comparative results of quantitative variables. The ends of the whiskers of the boxplots represent the minimum and maximum of all data. The bottom and top of the box represent the 25th and 75th percentile respectively, and the band near the middle of the box, the 50th percentile.

To establish the relationship between 24SNa increments and ISSD, Receiver Operating Characteristic ROC curves were used, calculating the area under the curve (AUC). Different cut-off points of ISSD linked to the SNa increase were obtained. Sensitivity (SS), specificity (SP), positive predictive value (PPV) and negative predictive value (NPV) were then calculated from the cut-off points selected by the researchers. Multivariate and univariate logistic regression analysis was performed and odd ratios (OR) were calculated to complement the association study between the ISSD and the 24SNa increase. The multivariable model included factors shown as statistically significant in the univariate analysis. A two-tailed *p* value < 0.05 was considered to be statistically significant. Ninety-five percent confidence intervals (95% CI) were calculated when applicable. Statistical analysis was performed using SPSS version 25 (IBM Corp., Armonk, NY, USA).

The study complied with accepted standards of good clinical practice, according to the Helsinki declaration. The research guidelines of the local committee were met and were known by all authors. The study did not require informed consent, given its retrospective nature.

## 3. Results

A total of 30 cases of patients with HH were analyzed. The median age of patients was 72 years (60–80), 15 (50%) were women. A majority of patients (23/30: 77%) had at least one risk factor for the development of ODS. The median pre-treatment SNa was 128 mmol/L (125–130). Thirteen (43%) patients had been receiving diuretics before hyponatremia was diagnosed. Eleven patients (37%) had a history of treatment with at least one of the following drugs: an angiotensin-converting enzyme inhibitor, an angiotensin-receptor blocker, or a mineralocorticoid-receptor antagonist. Thirteen patients (43%) had signs of infection coinciding with the hyponatremic episode. Nine patients presented with a gastrointestinal infection, two with pneumonia, and two with sepsis of unknown origin. In all patients, serum glycemia was consistently below 150 mg/dL, and correction of SNa for glycemia was not required. Other baseline patient characteristics are described in Table 1.

**Figure 1:**
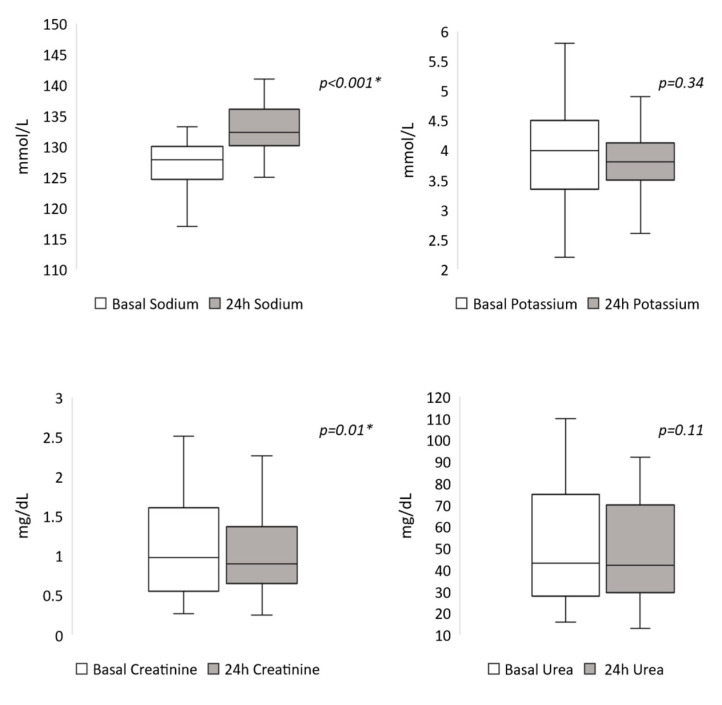
Evolution of serum biochemical parameters after 24 h of isotonic saline solution administration. * *p* < 0.05.

The median ISSD administered was 25 mL/kg/24 h (21–33). The 24-h levels of biochemical parameters after ISS administration were as follows: 132 mmol/L (130–136) for SNa, 3.8 mmol/L (3.5–4.1) for SK, 0.9 mg/dL (0.65–1.37) for serum creatinine, and 42 mg/dL (29.5–70) for serum urea. Comparative evolution of these parameters at 24 h are illustrated in Figure 1.

In 10 (33%) patients, the 24SNa showed a ∆ ≥ 8 mmol/L after a median ISSD of 32 mL/kg/24 h (29–37). In 15 (50%) patients, the 24SNa exhibited a ∆ ≥ 6 mmol/L after a median ISSD of 31 mL/kg/24 h (25–33). In 11 (48%) of the 23 patients with at least one risk factor for ODS, the 24SNa showed a ∆ ≥ 6 mmol/L, following a median ISSD of 31 mL/kg/24 h (23–37).

The 24SNa had a ∆ < 4 mmol/L in nine patients (30%), following the administration of a median ISSD of 20 mL/kg/24 h (14–22). In eight of these nine patients, the administered dose was ≤23 mL/kg/24 h.

**Figure 2:**
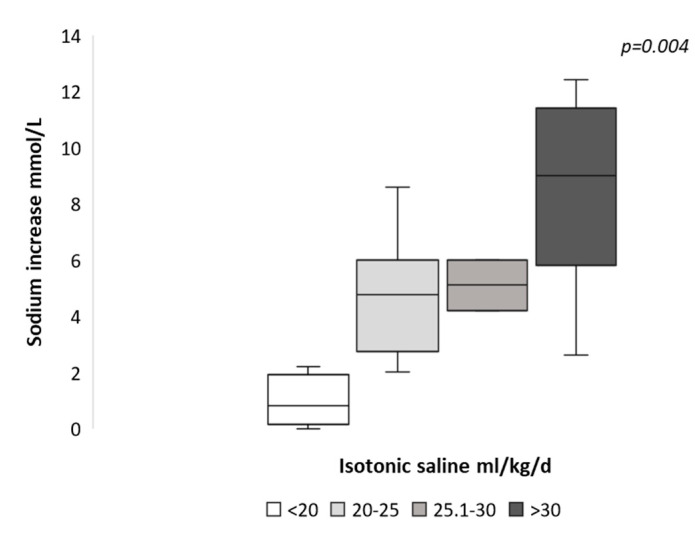
Median 24-h serum sodium (SNa) increments according to isotonic saline solution doses.

Patients receiving a larger volume of ISS had a higher 24SNa increment (Figure 2). There was a positive correlation between the ISSD and the 24SNa rise (Pearson correlation coefficient of +0.65, *p* = 0.001).

Excluding ISSD, the baseline SNa level was the only variable found to be associated with the 24-h SNa increase, and was lower in patients exhibiting a 24SNa ∆ ≥ 8 mmol/L (median 125 mmol/L (122–127)) as compared to those who did not (median 129 mmol/L (127–131), *p* = 0.044). The remaining univariate analyses performed for the entire group of patients are shown in Table 2.

Univariate logistic regression analysis revealed that for each mL/kg/24 h of ISS administered, the OR for a 24SNa ∆ ≥ 8 was 1.19 (95% CI: 1.036–1.368, *p* = 0.014), and of 1.16 (95% CI: 1.026–1.312, *p* = 0.018) for a 24SNa ∆ ≥ 6 in the entire group of patients. In those with ODS risk factors, the OR for a 24SNa ∆ ≥ 6 was 1.13 (95% CI: 1.007–1.278, *p* = 0.038).

In the entire group of patients, the AUC of ISSD in the ROC curve was 0.815 (95% CI: 0.661–0.969, *p* = 0.006) for a 24SNa ∆ ≥ 8 and of 0.791 (95% CI: 0.616–0.966, *p* = 0.007) for a 24SNa ∆ ≥ 6. In the group of patients with ODS risk, the AUC of ISSD was 0.788 (95% CI: 0.588–0.988, *p* = 0.019) for a 24SNa ∆ ≥ 6. Table 3 shows the cut-off points selected for SS, SP, PPV and NPV

Multivariate logistic regression analysis using forward Wald’s step method permitted determination of ORs for different ISSDs, in relation with the 24-h SNa increment, according to the cut-off points derived from the ROC curves, as given in Table 3. Based on these results, the ISSDs that permit reaching the goal of correction of SNa stipulated in our study are between 23–30 mL/kg/24 h.

## 4. Discussion

The current study has found that, in patients with HH, the 24SNa increment is directly related to the ISSD employed for therapy. This was the case both for the entire group of patients studied, as well as for those with at least one risk factor for the development of ODS.

Clinical guidelines and expert opinions recommend the administration of isotonic saline/NaCl 0.9% for the treatment of patients with HH, while indicating the need for close follow-up, to avoid overcorrection of serum sodium levels [1,2,3,19,22]. However, the ISSD necessary to induce an adequate elevation of SNa levels without provoking overcorrection is unknown. Sahay M. and Sahay R. [3] suggest a NaCl 0.9% dose of 0.5–1 mL/kg/h, without specifying the duration of therapy, nor the probability of therapeutic success or overcorrection. Based on the results of the current study, the dose recommended by Sahay M. and Sahay R., when administered over a 24-h period to a subject weighing 70 kg (14–24 mL/kg/24 h), would have a low risk for overcorrection. However, there would be a high risk for undercorrection, since in our study, eight of nine patients receiving a similar dose (≤23 mL/kg/24 h) did not attain the minimum therapeutic goal of a 4 mmol/L 24SNa rise [2,22].

Among measures proposed to avoid overcorrection during ISS administration are the measurement of SNa every 4–6 h as well as the monitoring of diuresis [1,2,23]. Desmopressin administration can also be associated [9,14,24,25,26]. However, in some hospital settings, all or parts of these recommendations could be difficult to apply. Therefore, if the mainstay of therapy of HH is ISS administration, an estimation of a dose with a high probability of achieving the stated goals is desirable. We have found that an infusion rate between 23–30 mL/kg/24 h of ISS would comply with these requisites.

Although a younger age [10,27], female sex [10], a low weight [10,28], malnutrition [9], hypokalemia as well as its treatment with KCl [2,9,17,29], and a lower baseline SNa [10,14,28] have all been associated with an increased risk for overcorrection, the current study has only found a significant association between a low baseline SNa and a 24-h SNa ∆ ≥ 8, with a tendency detected in the rest of variables analyzed. We cannot, however, rule out that some of these parameters would be associated with a significant SNa rise if our study population had been larger. In any event, our findings suggest that the principal determinant of the 24-h SNa rise is the dose of ISS itself.

The main limitation of this research is the small number of cases studied. An additional limitation is its retrospective methodology.

A strong point of the current study is the correct volemic classification of patients, based on various parameters that adhere to current scientific evidence [2,16,19,20]. Without a correct diagnosis of hypovolemia, its therapy simply cannot be evaluated [2,9,17,18,30]. Furthermore, essential clinical information, such as recent patient weight and the SNa level precisely 24 h after the start of ISS therapy, were available in all cases. The direct clinical relevance of the results is an additional strong point.

In conclusion, the ISS dose is directly related to the 24-h SNa rise following initiation of infusion. Based on our results, we recommend an ISS dose of 23 to 30 mL/kg/24 h to achieve an increase in SNa between 4–7 mmol/L over the first 24 h of therapy in patients with HH. Further prospective studies with a larger number of patients should be undertaken to confirm these results.

## Figures and Tables

**Table 1:** Characteristics of the patients of the study.

*N* = 30 (100%)	
Age, years	72 (60–80)
Sex female, *n* (%)	15 (50%)
**Clinical markers of hypovolemia**	
Blood pressure ≤ 90/60 mmHg, *n* (%)	7 (23%)
Heart rate ≥ 90 bpm, *n* (%)	12 (40%)
Decreased internal jugular vein pulse height (*n* = 22), *n* (%)	22 (100%)
Orthostatic symptoms (*n* = 20), *n* (%)	14 (70%)
**Biochemical serum parameters**	
Sodium mmol/L	128 (125–130)
Potassium mmol/L	4 (3.4–4.5)
Creatinine mg/dL	0.98 (0.55–1.61)
Urea mg/dL	43 (27.7–75)
**Risk Factors for Osmotic demyelination syndrome, *n* (%)**	23 (78%)
Malnutrition, *n* (%)	18 (60%)
Hypokalemia, *n* (%)	9 (30%)
Alcoholism, *n* (%)	2 (7%)
Liver disease, *n* (%)	1 (3%)
**Location of the patients**	
Emergency room, *n* (%)	16 (53%)
Hospitalization ward, *n* (%)	14 (47%)
**Type of isotonic saline solution**	
NaCl 0.9%, *n* (%)	25 (83%)
NaCl 0.81%, *n* (%)	5 (17%)
**Etiology of hypovolemia**	
Unknown, *n* (%)	8 (27%)
Gastrointestinal losses, *n* (%)	13 (43%)
Urinary losses, *n* (%)	17 (57%)
Blood loss *n* (%)	2 (7%)

Quantitative variables are given as median and (interquartile range).

**Table 2:** Comparative analysis of the clinical variables studied as function of 24-h SNa increase.

	24-h SNa ∆ ≥ 8 mmol/L			24-h SNa ∆ ≥ 6 mmol/L		
	Yes (*n* = 10)	No (*n* = 20)	*p* *	Yes (*n* = 15)	No (*n* = 15)	*p* *
**Female, %**	70	40	0.12	67	33	0.68
**Male, %**	30	60		33	67	
**Age, years**	72 (47–84)	75 (60–79)	0.681	72 (51–83)	72 (60–77)	0.967
**Weight, kg**	53 (49–61)	61 (53–72)	0.067	58 (49–65)	58 (53–72)	0.187
**Basal serum sodium, mmol/L**	125 (122–127)	129 (127–131)	0.044 *	125 (122–131)	129 (127–130)	0.067
**Basal serum sodium ≤ 120 mmol/L, %**	10	10	1	13.3	6.7	1
**Risk factors for osmotic demyelination syndrome, %**	80	75	0.76	73.3	80	0.66
Malnutrition, %	60	60	1	53	67	0.45
Hypokalemia, %	30	30	1	27	34	1
Alcoholism, %	0	10	0.3	0	13	0.14
Liver disease, %	0	5	0.47	0	7	0.3
**Duration of hyponatremia**						
Acute, %	30	15	0.372	33	7	0.169
Chronic, %	70	85		67	93	
**Location of patients**						
Emergency room, %	50	45	1	60	33	0.272
Hospitalization ward, %	50	55		40	67	
**Associated potassium chloride treatment, %**	30	30	1	27	33	1
**Type of isotonic saline solution**						
NaCl 0.9%, %	90	80	0.64	93	73	0.33
NaCl 0.81%, %	10	20		7	27	
**Isotonic saline solution dose, mL/kg/24 h**	32 (29–37)	23 (20–30)	0.005 *	31 (25–33)	21 (17–29)	0.006 *

Quantitative variables are given as median and (interquartile range).* *p* < 0.05. SNa ∆: variation in serum sodium.

**Table 3:** Sensitivity, specificity, positive predictive value, negative predictive value and odd ratio of the different isotonic saline solution doses for 24-h SNa ∆ ≥ 8 and ≥ 6 mmol/L.

	Sensitivity (95% CI)	Specificity (95% CI)	Positive Predictive Value (95% CI)	Negative Predictive Value (95% CI)	Odd Ratio (95% CI) *	*p* *
**SNa ∆ ≥ 8**						
**Isotonic saline dose:**						
≥30 mL/kg/24 h	80% (50–100)	80% (60–100)	67% (36–98)	89% (72–100)	16 (2.5–95.1)	0.004 *
≥22 mL/kg/24 h	100% (95–100)	45% (21–69)	48% (24–71)	100% (94–100)	-	-
**SNa ∆ ≥ 6**						
**Isotonic saline dose:**						
≥29 mL/kg/24 h	67% (39–94)	80% (55–100)	77% (50–100)	71% (46–95)	13 (2.2–72.1)	0.007 *
≥22 mL/kg/24 h	100% (97–100)	60% (32–88)	71.4% (50–93)	100% (94–100)	-	-
SNa ∆ ≥ 6 **						
Isotonic saline dose: ≥29 mL/kg/24 h	73% (42–100)	75% (46–100)	73% (42–100)	75% (46–100)	8 (1.2–51.5)	0.029 *

* *p* < 0.05. Multivariate logistic regression analysis including parameters with statistical significance in the univariate analysis. ** Calculated in patients with risk factors for osmotic demyelination syndrome. SNa ∆: variation in serum sodium.
